# Epigenetic Silencing of Host Cell Defense Genes Enhances Intracellular Survival of the Rickettsial Pathogen *Anaplasma phagocytophilum*

**DOI:** 10.1371/journal.ppat.1000488

**Published:** 2009-06-19

**Authors:** Jose C. Garcia-Garcia, Nicole C. Barat, Sarah J. Trembley, J. Stephen Dumler

**Affiliations:** 1 Department of Pathology, The Johns Hopkins University School of Medicine, Baltimore, Maryland, United States of America; 2 Department of Molecular and Comparative Pathobiology, The Johns Hopkins University School of Medicine, Baltimore, Maryland, United States of America; Yale University School of Medicine, United States of America

## Abstract

Intracellular bacteria have evolved mechanisms that promote survival within hostile host environments, often resulting in functional dysregulation and disease. Using the *Anaplasma phagocytophilum*–infected granulocyte model, we establish a link between host chromatin modifications, defense gene transcription and intracellular bacterial infection. Infection of THP-1 cells with *A. phagocytophilum* led to silencing of host defense gene expression. Histone deacetylase 1 (HDAC1) expression, activity and binding to the defense gene promoters significantly increased during infection, which resulted in decreased histone H3 acetylation in infected cells. HDAC1 overexpression enhanced infection, whereas pharmacologic and siRNA HDAC1 inhibition significantly decreased bacterial load. HDAC2 does not seem to be involved, since *HDAC2* silencing by siRNA had no effect on *A. phagocytophilum* intracellular propagation. These data indicate that HDAC up-regulation and epigenetic silencing of host cell defense genes is required for *A. phagocytophilum* infection. Bacterial epigenetic regulation of host cell gene transcription could be a general mechanism that enhances intracellular pathogen survival while altering cell function and promoting disease.

## Author Summary

Although the main function of defense cells is to eliminate invading infections, some intracellular bacterial pathogens manage to turn defense cells into suitable hosts for bacterial propagation. In doing so, intracellular pathogens dysregulate host cell function and cause disease. With genomic and metabolic resources thousands of times more limited than the host's, intracellular bacteria have evolved very efficient mechanisms to globally subvert the host defense. Here, we define a mechanism by which the intracellular pathogen *Anaplasma phagocytophilum* globally inhibits host cell defenses by affecting mechanisms of epigenetic control of defense gene expression. Silencing or inhibition of the host protein HDAC1 has a negative effect on intracellular bacterial replication, whereas HDAC1 overexpression leads to defense gene silencing and facilitates intracellular bacterial survival. This study not only provides new insight into a mechanism of host cell subversion, but also identifies a potential target for future development of novel therapeutic intervention strategies.

## Introduction

Intracellular pathogens, through a long-standing association with host cells, have evolved mechanisms that allow survival within the often hostile environment of their hosts [1]. These mechanisms usually result in dramatic transcriptional changes in infected host cells and in dysregulation of cell functions that potentially lead to disease. Global analysis of mammalian gene expression in response to intracellular bacteria has led to the identification of major pathways affected during infection [2]. Due to the limited genetic and metabolic resources of intracellular bacteria, these pathogens likely evolved global and efficient mechanisms for host cell gene regulation. While signaling pathways and transcriptional regulators often act on a limited subset of genes, epigenetic regulators tend to more globally control gene expression, and impact major cellular processes such as cell cycle progression and cell differentiation. Dysregulation of epigenetic control mechanisms often leads to dramatic phenotypic changes.

Reversible histone acetylation is a key epigenetic regulator of chromatin structure and gene expression, in combination with other posttranslational modifications. These patterns of histone modification are maintained by histone modifying enzymes such as histone deacetylases (HDAC). Disruption of HDAC activity with inhibitors or by siRNA affects expression of up to 10% of the genes in different cell types [3]–[6]. Global HDAC-mediated transcriptional changes can have a concomitant effect on cell function – an epigenetic mechanism often exploited by viruses to promote infection [7]–[9]. Recent reports show that intracellular bacteria manipulate host cell epigenetics to facilitate infection as well [10]–[12].

The tick-transmitted rickettsial pathogen *Anaplasma phagocytophilum*, causative agent of human granulocytic anaplasmosis, is one of only four bacteria known to survive and propagate within human neutrophils and their bone marrow progenitors. Neutrophils are generally considered unsuitable host cells for intracellular bacteria because they are short-lived and are primary defense cells equipped with diverse antimicrobial mechanisms. Major neutrophil functions are altered with *A. phagocytophilum* infection, which ultimately results in clinical disease. Processes such as oxidative burst, apoptosis and phagocytosis are inhibited or delayed by *A. phagocytophilum* infection [13]–[15], while degranulation and cytokine/chemokine production are activated [16]–[18]. These dramatic alterations in host cell function can be explained at least in part by *A. phagocytophilum*-induced host transcriptional changes [19]–[22]. We have recently shown that the *A. phagocytophilum* effector protein AnkA is translocated into the host nucleus, where it interacts with host chromatin to affect gene transcription [23]–[25]. However, global mechanisms leading to *A. phagocytophilum*-induced transcriptional changes remain poorly defined.

In this report, we investigate the hypothesis that global down-regulation of key host defense genes is critical for *A. phagocytophilum* intracellular infection and propagation. We establish a link between intracellular bacterial survival/growth and changes in host transcription and function known to be involved in *A. phagocytophilum* pathogenesis. These data suggest a global epigenetic mechanism by which bacterial pathogens interact with and control host cells.

## Results

### Defense gene expression is down-regulated in *A. phagocytophilum*-infected cells

**Figure 1:**
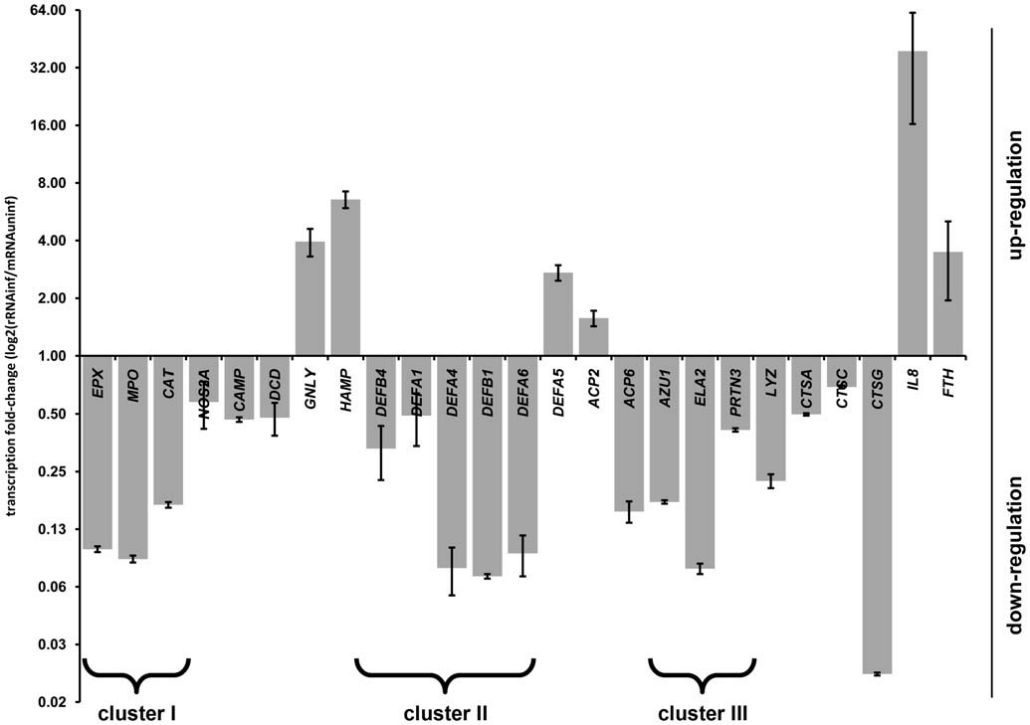
Down-regulation of host defense genes with *A. phagocytophilum* infection of THP-1 cells. RNA was extracted from infected and uninfected THP-1 cells 48 hours post-infection and expression of defense genes was quantitated by qRT-PCR. *IL8* and *FTH* expression were used as up-regulation controls. Gene expression changes were expressed as transcription fold-change in infected cells with respect to uninfected cells. Numbers <1 denote down-regulation and >1 indicate up-regulation.

Previous studies suggest that *A. phagocytophilum* infection down-regulates the expression of key host defense genes such as *CYBB*, *RAC2*, *MPO* and *BPI* [26]. Expression of 23 defense genes, including genes encoding for antimicrobial peptides as well as genes involved in enzymatic and oxidative defense mechanisms, was compared by quantitative RT-PCR (qRT-PCR). Expression of 19 of these defense genes was down-regulated during infection of THP-1 cells with *A. phagocytophilum* (Fig. 1), whereas expression of *IL8* and *FTH*, two genes known to be up-regulated during *A. phagocytophilum* infection of granulocytes, was increased. Notably, the *EPX* and *MPO* genes, which form a gene cluster in the genome (17q23), were down-regulated, as were most of the defensins, which comprise a gene cluster on chromosome 8p23, and the genes *AZU1*, *ELA2* and *PRTN3*, which form a third defense gene cluster on chromosome 19p13 (Fig. 1). From this and previously published data, it can be concluded that defense gene expression is globally down-regulated with *A. phagocytophilum* infection, permitting sustained inhibition of antimicrobial defense and facilitating establishment of intracellular infection.

### *A. phagocytophilum* infection affects defense gene chromatin structure

**Figure 2:**
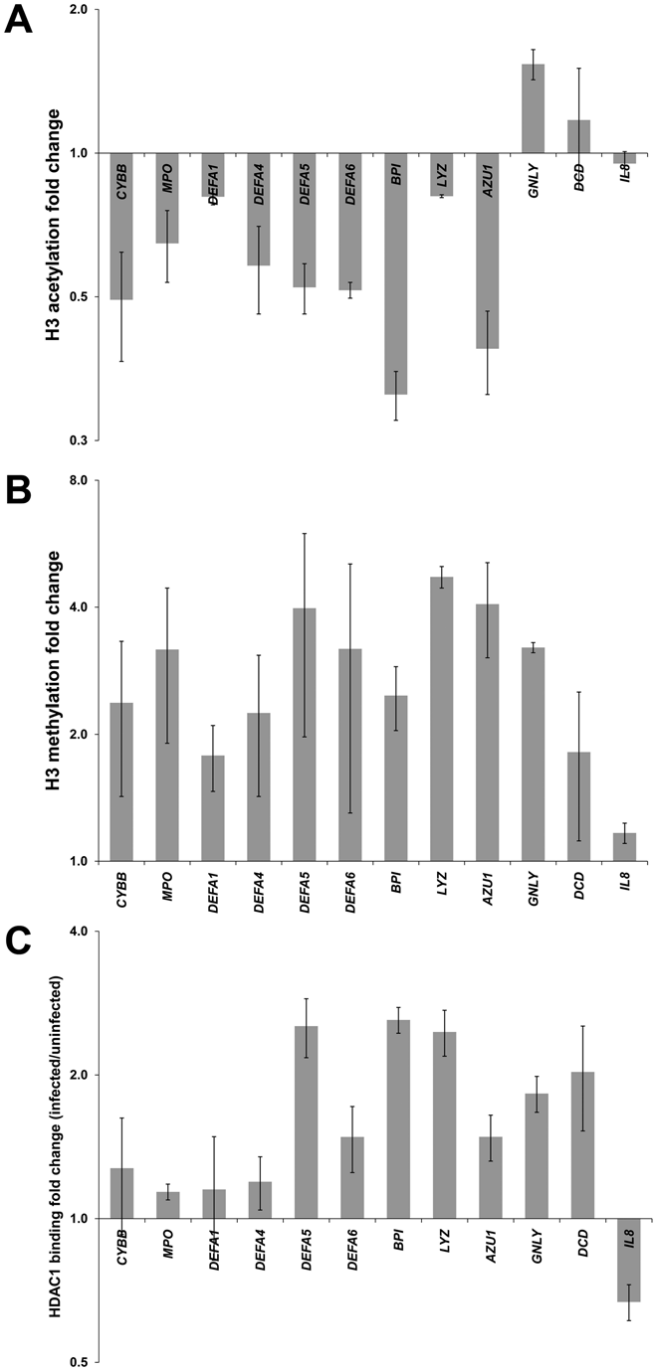
The pattern of histone post-translational modifications and HDAC1 binding in the defense gene promoters is affected by *A. phagocytophilum* infection of THP-1 cells. Chromatin from infected and uninfected THP-1 cells was prepared 48 hours post-infection. The histone modification pattern and HDAC1 binding at the defense gene promoters was analyzed by ChIP using antibodies specific for (A) Ac-H3, (B) Me-H3 and (C) HDAC1. Immunoprecipitated DNA fragments were quantitated by qPCR using primers specific for each promoter region. Changes were expressed as the ratio of immunoprecipitated chromatin target from infected to uninfected cells enriched with respect to input chromatin.

Silencing of genes or gene clusters is often associated with changes in chromatin structure mediated by epigenetic regulators. In order to identify possible chromatin alterations associated with *A. phagocytophilum* infection, a chromatin immunoprecipitation (ChIP) approach was used to study alterations in histone H3 diacetylation (Ac-H3) and monomethylation (Me-H3) patterns at defense gene promoters (Fig. 2). A decrease in the Ac-H3 with *A. phagocytophilum* infection was observed in 9 out of 11 defense gene promoters (Fig. 2A), consistent with decreased transcriptional activity at these loci during infection (Fig. 1A, B). Slightly increased Ac-H3 was observed in the *GNLY* and *DCD* promoters, whereas no changes were observed in the *IL8* promoter, which was used as a control. *IL8*, unlike defense genes, is upregulated by signal transduction pathways activated with infection. A concomitant increase in Me-H3 was observed for all genes except *IL8*. This difference was not significant for *DEFA1*, *DEFA6* and *DCD*. These results suggest that silencing of most key host defense genes with *A. phagocytophilum* infection could occur by epigenetic changes in chromatin structure and histone post-translational modification patterns.

Since histone deacetylases play a crucial role in regulating histone post-translational modifications, we next investigated changes in HDAC1 binding to the promoter regions of host defense genes during *A. phagocytophilum* infection (Fig. 2C). After infection, HDAC1 binding was increased to the promoters of all 11 defense genes analyzed. HDAC1 acts as a transcriptional repressor and its increased association with defense gene promoters upon *A. phagocytophilum* infection suggests a role for HDAC1 in defense gene silencing.

### Deacetylase expression and activity are increased with *A. phagocytophilum* infection

**Figure 3:**
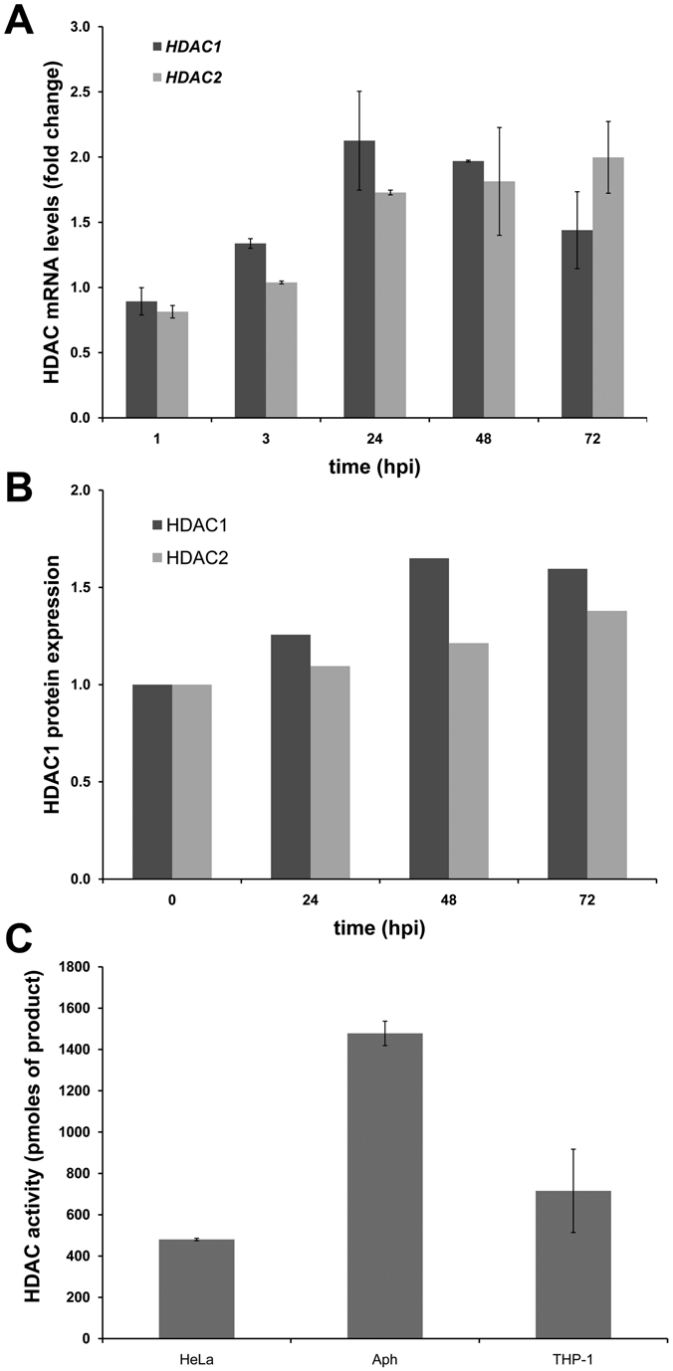
HDAC expression and activity is increased by *A. phagocytophilum* infection. (A) RNA from infected and uninfected cells was extracted and *HDAC1* and *HDAC2* expression was quantitated by qRT-PCR. Transcription fold-change with respect to uninfected cells was calculated. (B) HDAC1 and HDAC2 in infected and uninfected THP-1 cells were detected by immunoblotting. Band intensity was determined by densitometric analysis and protein expression level changes with respect to the initial expression level in uninfected cells were calculated. Samples were normalized for β-actin content. The example shown is representative of 3 separate experiments with similar results. (C) HDAC activity in nuclear extracts of infected and uninfected cells at 48 hpi was determined using a fluorescent assay kit. Nuclear extract from HeLa cells was used as control.

Since histone deacetylases play a crucial role in regulating histone post-translational modifications, we next investigated changes in HDAC expression and activity associated with *A. phagocytophilum* infection. Gene expression analysis of *HDAC1* and *HDAC2* in *A. phagocytophilum*-infected cells by qRT-PCR showed a transient increase in *HDAC1* expression that peaked within 24 hours post-infection, whereas *HDAC2* transcription steadily increased over time (Fig. 3A). Following the initial increase, *HDAC1* mRNA levels decreased by 72 hours, similar to the profile observed in *A. phagocytophilum*-infected HL-60 cells (not shown), suggesting HDAC1-mediated transcriptional autorepression. HDAC1 protein levels started to increase within 24 hours after *A. phagocytophilum* infection, increasing greater than 1.5-fold by 48 hours and moderately decreasing thereafter (Fig. 3B). HDAC2 protein levels steadily increased over time with kinetics similar to that of *HDAC2* gene transcription (Fig. 3A, B). HDAC activity was also significantly increased in infected cells 48 h post-infection (Fig. 3C). Taken together, these results indicate that HDAC1 and HDAC2 expression is increased upon *A. phagocytophilum* infection by mechanisms regulated at least in part at the transcriptional level. Three human strains of *A. phagocytophilum* from North America and Europe showed similar abilities to increase HDAC expression and activity (Figure S1).

### Increased HDAC activity down-regulates defense gene expression and promotes *A. phagocytophilum* infection of THP-1 cells

**Figure 4:**
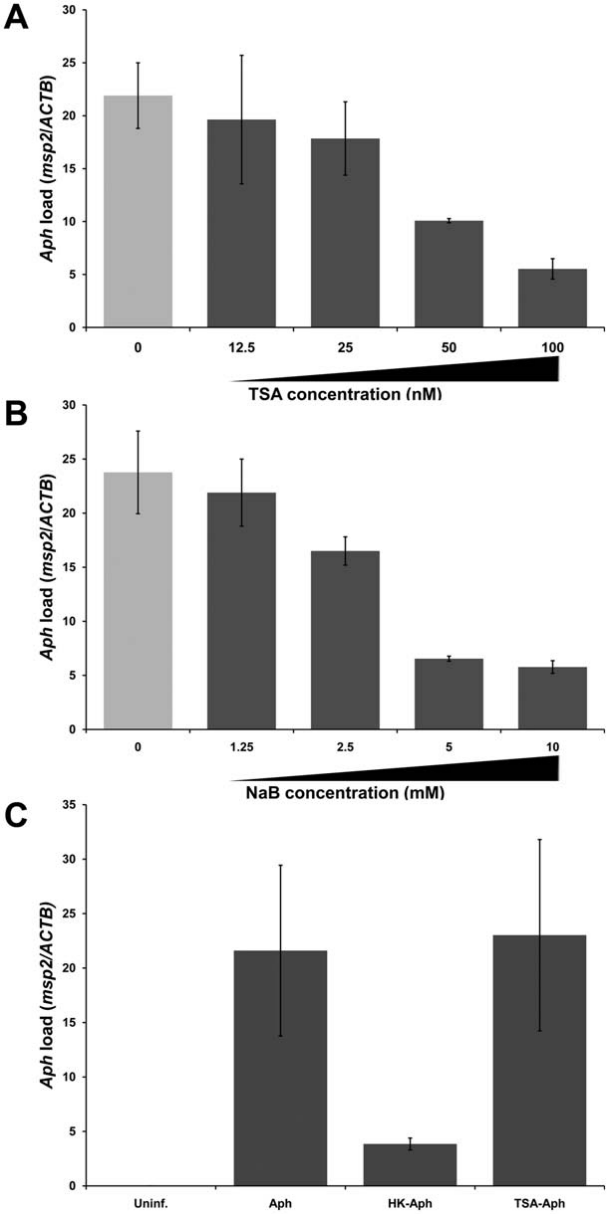
HDAC inhibition impairs the ability of *A. phagocytophilum* to propagate intracellularly. THP-1 cells were infected with cell-free *A. phagocytophilum* and incubated with or without the HDAC inhibitors TSA and sodium butyrate. Twenty-four hours post infection, cells were collected and *A. phagocytophilum* infection level was determined by qPCR. The first point represents infection level in the absence of HDAC inhibitor. Cells were incubated with (A) TSA or (B) sodium butyrate. Infection levels were normalized for β-actin gene content to account for differences in the number of viable cells. (C) Cell-free *A. phagocytophilum* preincubated with 100 nM TSA for 2 hours prior to infection was used to infect THP-1 cells as described. *A. phagocytophilum* load was determined by qPCR 24 hours later.

To determine whether increased HDAC1 expression is required for *A. phagocytophilum* to establish successful intracellular infection, HDAC activity was inhibited prior to infection using the HDAC inhibitors trichostatin A (TSA) and sodium butyrate. *A. phagocytophilum* infection was reduced in a dose-dependent manner by both HDAC inhibitors (Fig. 4A, B). *A. phagocytophilum* infection was not significantly reduced at lower doses of either inhibitor, but at higher doses *A. phagocytophilum* infection was significantly reduced (p<0.05). Preincubation of *A. phagocytophilum* with TSA did not affect its ability to infect THP-1 cells (Fig. 4C), ruling out HDAC inhibitor toxicity toward *A. phagocytophilum*.

**Figure 5:**
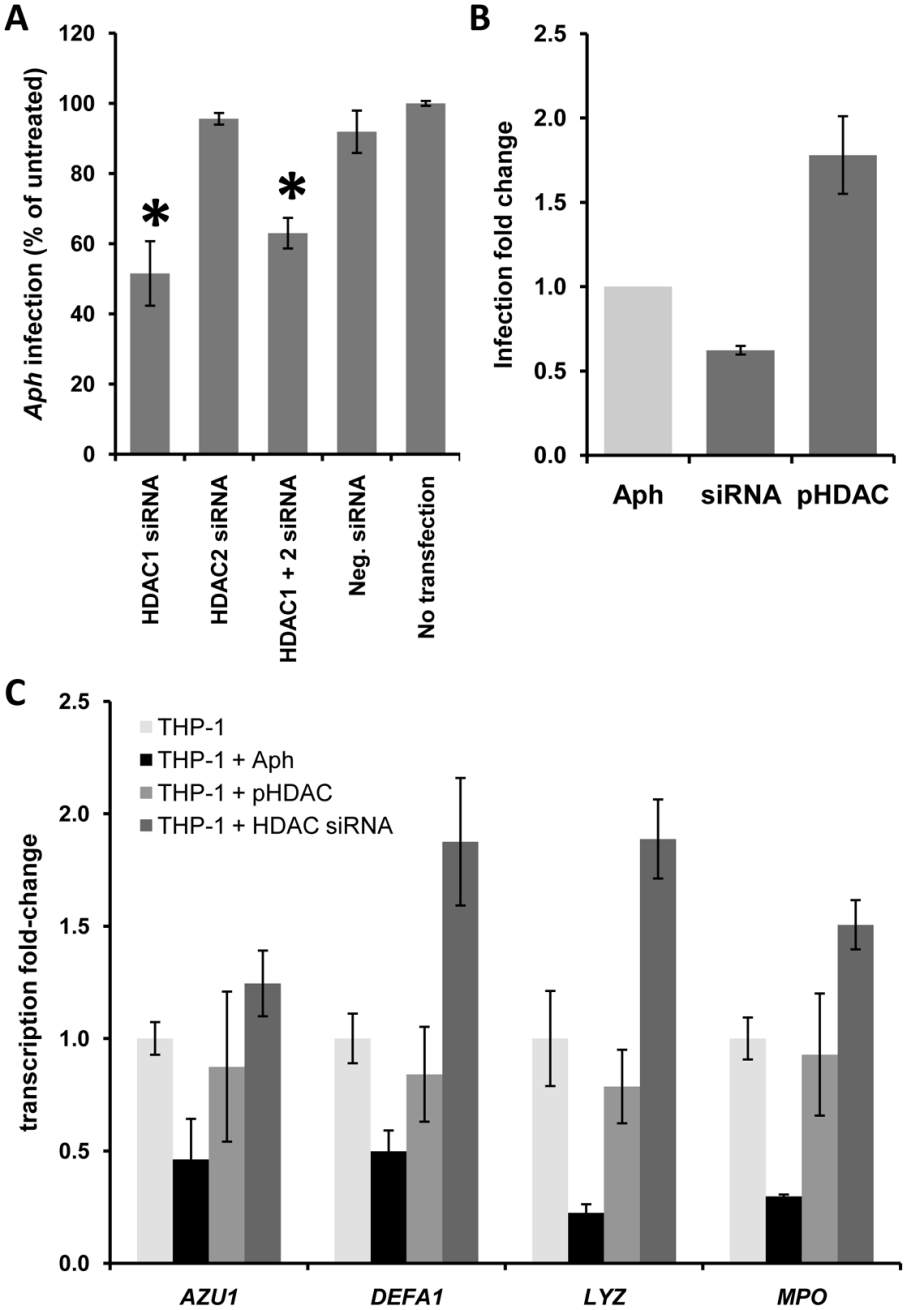
HDAC1 silences defense gene expression and facilitates *A. phagocytophilum* infection of THP-1 cells. (A) THP-1 cells were transfected with *HDAC1* or *HDAC2* siRNA and were infected 24 hours after transfection with cell-free *A. phagocytophilum*. The *A. phagocytophilum* load was determined 24 h after infection by qPCR and normalized to infection levels in non-transfected cells. (B) THP-1 cells were transfected with *HDAC1* siRNA for silencing or with pHDAC1-FLAG plasmid to overexpress HDAC1 and were infected 24 hours after transfection with cell-free GFP-expressing HGE-1 strain of *A. phagocytophilum*. The percent of infected cells was determined 24 h after infection by flow cytometry and normalized to infection levels in untreated cells. (C) Expression of defense genes by THP-1 cells infected with *A. phagocytophilum*–infected or transfected with pHDAC1-FLAG plasmid or *HDAC1* siRNA was determined by qRT-PCR. Gene expression was normalized to housekeeping genes and to the level of expression of untreated, uninfected THP-1 cells.

Because TSA inhibits not only HDAC1 but also other class I acetylases, HDAC1 expression was specifically targeted using siRNA. *HDAC1* silencing resulted in a significant reduction in *A. phagocytophilum* load in infected THP-1 cells, whereas *HDAC2* silencing did not affect *A. phagocytophilum* infection (Fig. 5A). The possible role of other deacetylases and the lower efficiency of siRNA compared to pharmacological inhibition, may explain the less dramatic effect of *HDAC1* silencing on *A. phagocytophilum* infection as compared to HDAC inhibition with TSA or sodium butyrate. On the other hand, the infectious load of THP-1 cells transfected with an HDAC1-expressing plasmid increased (Fig. 5B), indicating that HDAC1 expression enhances *A. phagocytophilum* infection and propagation. To confirm that HDAC1 plays a direct role in silencing host defense genes, the expression of four defense genes was analyzed in THP-1 cells transfected with *HDAC1* siRNA or pHDAC1-FLAG plasmid. Expression of *DEFA1*, *AZU1*, *LYZ* and *MPO* was upregulated by siRNA *HDAC1* silencing, whereas *A. phagocytophilum* infection and HDAC1 overexpression led to decreased gene expression (Fig. 5C). These data confirm the role of HDAC1 in host defense gene silencing and host cell conditioning for intracellular survival.

## Discussion

The intracellular environment is hostile for bacteria that propagate within defense cells such as macrophages, monocytes and granulocytes. These cells are equipped with an arsenal of antimicrobial molecules that target pathogens using diverse mechanisms, including generation of reactive oxygen intermediates and secretion of antimicrobial peptides and enzymes. With a cytoplasm and a genome over a thousand times smaller than those of the host cell, *A. phagocytophilum* and other intracellular bacteria must require efficient mechanisms for survival and persistence within the host cell by inducing sustained changes in host cell function. The data presented here point to a mechanism, conserved among human isolates of *A. phagocytophilum*, for control of host cell gene expression and function based on host cell epigenetic changes. Increased HDAC expression in *A. phagocytophilum*-infected cells results in accumulation of deacetylated histones. These changes in the pattern of histone posttranslational modifications likely have a direct effect on gene expression by affecting chromatin structure, leading to a highly compact chromatin conformation with limited access to transcriptional activators. Importantly, defense genes are often organized in chromosomal clusters that permit coordinate expression and regulation by changes in chromatin organization [27]. Moreover, increased HDAC expression could also directly down-regulate the expression of genes which are transcriptionally repressed by HDACs, such as *CYBB* [28]. The data presented here suggest a new mechanism mediated by HDACs by which *CYBB* and other defense genes can be silenced by *A. phagocytophilum* infection. Other bacteria seem to take advantage of similar epigenetic mechanisms of control of host cell function. *Listeria monocytogenes*, *Clostridium perfringens* and *Streptococcus pneumonia* induce H3 dephosphorylation as well as H4 deacetylation during infection, which correlate with down-regulated expression of a subset of host genes including defense genes [11]. Similarly, mycobacterial infection of human cells results in host gene silencing using a mechanism that involves HDAC complex formation and histone deacetylation [29]. Host cell epigenetics, bacterial effectors such as the AnkA protein, signaling pathways and other mechanisms of transcriptional regulation likely contribute to the overall regulation of host cell gene expression and function.

The data presented here provide evidence that *A. phagocytophilum* infection leads to modified host cell gene transcription and phenotype by epigenetically altering host chromatin in regions that play a regulatory role in gene expression – a global mechanism for control of eukaryotic host cell function by intracellular bacteria. Other bacteria that closely interact with host cells, whether intracellular or not, may use similar mechanisms for manipulating host cell function. Further study of this host control mechanism could define prokaryotic effectors associated with this process and facilitate development of new strategies for the prevention and treatment of infections caused by bacteria with intimate host cell associations.

## Materials and Methods

### Cell lines and cell culture

The acute monocytic leukemia THP-1 (ATCC CCL-240) cell line was used in this study because it supports *A. phagocytophilum* growth and can be readily transfected with siRNAs and plasmids as described below. THP-1 cells were grown in RPMI medium containing 10% FBS in a humidified incubator at 37°C with 5% CO_2_. Cell density was kept <5×10^5^ cells/mL by diluting with fresh medium every three days. The cell permeable HDAC inhibitors TSA and sodium butyrate were used at concentrations of 10–800 nM and 1–10 mM, respectively. The proportion of infected cells was determined by microscopic examination of LeukoStat-stained cells.

### *Anaplasma phagocytophilum* culture and isolation

A GFP-expressing HGE-1strain of *A. phagocytophilum* [30], as well as the Webster, Slovenia and HZ strains were used in this study. Infected cells were grown until >90% of the cells were infected. Uninfected THP-1 cells were used to adjust the infection level to 10% as needed. To isolate cell-free *A. phagocytophilum*, 2×10^7^ infected cells were collected by centrifugation at 500×g for 5 min. Bacteria were released by lysis using a 25⅝G syringe needle. Nuclei and cell debris were removed by low speed centrifugation (1,000×g, 5 min) and *A. phagocytophilum* organisms were collected from the supernatant by centrifugation at 14,000×g for 10 min. The bacterial preparation was finally resuspended in RPMI medium for *in vitro* infection of THP-1 cells at a multiplicity of infection (MOI) of 10 bacteria per cell. Infection levels were determined by microscopy examination of stained cells, by qPCR or by flow cytometry as described below.

### Histone extraction and analysis

Histones were isolated from *A. phagocytophilum*-infected and uninfected cells by acid extraction as described previously [31]. Briefly, nuclei from 2×10^7^ cells were obtained by hypotonic lysis. Histones were then extracted with 0.4 N H_2_SO_4_, TCA-precipitated and washed with acetone. To study changes in histone posttranslational modifications, equal amounts (1 µg) of isolated histones were separated in 15% SDS-PAGE and analyzed by immunoblotting using antibodies specific for Ac-H3 and Me-H3.

### Cell fractionation and immunoblotting

Nuclear extracts from 2×10^7^ *A. phagocytophilum*-infected or uninfected THP-1 cells were prepared using NE-PER Nuclear and Cytoplasmic Extraction Reagents (Pierce, USA) with protease inhibitors (PMSF and cOmplete, Roche, USA). Samples of the nuclear fractions were analyzed by immunoblotting to determine the presence of HDAC1 or HDAC2. Cytoplasmic fractions were used for normalization for β-actin content.

Approximately 1 µg total protein was electrophoresed in a 10% or 15% SDS-PAGE gel and transferred to a nitrocellulose membrane. The blot was blocked with 3% bovine serum albumin, and probed with 1∶2,000 HDAC1 rabbit polyclonal antibody (Sigma, USA), 4 µg/mL HDAC2 monoclonal antibody (Sigma, USA), 1∶100 Ac-H3 rabbit polyclonal antibody or 1∶100 anti-Me-H3 (Millipore, USA). Human β-actin was used for normalization. Membranes were incubated with goat anti-mouse or anti-rabbit alkaline phosphatase conjugate (KPL, USA) and developed using Immun-Star AP substrate (Bio-Rad, USA). Band intensities were determined by densitometry using the public domain, free software ImageJ, Image processing and analysis in Java (http://rsb.info.nih.gov/ij/).

### HDAC activity assay

Nuclear extracts from infected or uninfected THP-1 cells were prepared using NE-PER Nuclear and Cytoplasmic Extraction Reagents (Pierce, USA) as described above, and used to determine HDAC activity with the fluorescent HDAC assay kit (Active Motif, USA) following the manufacturer's instructions. Nuclear extracts were diluted 1∶3 in assay buffer and activity expressed as pmoles of fluorescent product formed after incubation at 37°C for 1 hour. Fluorescence intensity was measured using a Perkin Elmer Victor II plate reader with an excitation wavelength at 355 nm and emission at 460 nm.

### RNA isolation and quantitative RT-PCR

To study the level of gene expression, total RNA from approximately 2×10^6^ cells was purified using RNeasy RNA extraction Kit (Qiagen, USA). cDNA was synthesized using SuperScript Reverse Transcriptase Kit (Invitrogen, USA) and quantitative PCR analysis was performed using primer sets specific for genes encoding for *HDAC1*, *HDAC2* or for defense genes using a custom-built PCR array (Table S1). Real time PCR was performed using SYBR Green Supermix in an iQ5 Multicycler (Bio-Rad, USA). Primer sequences were taken from the qPrimerDepot database [32]. Expression levels were calculated using the comparative Ct method with the average Ct of human housekeeping genes *ACTB*, *B2M*, *GAPDH*, *HPRT1* and *RPL13A* as normalizer and uninfected cells as reference. The average±std. dev. of three experiments was calculated.

### Chromatin immunoprecipitation

Analysis of histone modification patterns in the 1,000 bp proximal promoter regions of defense genes was performed using ChIP followed by qPCR using promoter-specific primers. Infected THP-1 cells (10^8^) were cross-linked with formaldehyde at 1% final concentration for 15 min at room temperature. Cross-linking and chromatin isolation were performed as described [33]. Chromatin was sheared 4 times for 15 sec using a Branson Sonifier 250 (Branson, USA) at 1.5 constant output power. Histone-bound DNA was immunoprecipitated using Ac-H3 or Me-H3 -specific antibodies (Millipore, US). Input chromatin was normalized and uninfected THP-1 cells were used as control. A negative, isotype-matched antibody control ChIP was also included. A sample of total chromatin was used as a positive control and for normalizing. Immunoprecipitated DNA fragments were quantitated by qPCR using primer sets specific for the defense gene promoter regions. The relative enrichment of each DNA fragment was calculated from the difference of the Ct with respect to the negative antibody control ChIP and normalized to the total chromatin control. Experiments were repeated two times and the average of the two determinations was calculated.

### Plasmid and small interference RNA transfection

To overexpress HDAC1, 2×10^6^ THP-1 cells were nucleofected with 1 µg of pHDAC1-FLAG (Addgene plasmid 13820) purified using EndoFree Plasmid isolation kit (Qiagen, USA). Nucleofection was performed using Amaxa's nucleofector technology (Amaxa, Germany), Cell Line Nucleofector Kit V and program U-01. Cells transfected with plasmid vector pcDNA3.1 were used as control. Six hours after transfection, cells were infected with cell-free GFP-expressing *A. phagocytophilum* to assess the effect of HDAC1 overexpression on infection by flow-cytometry, expressed as changes in the proportion of infected THP-1 cells with respect to the control.

HDAC1 expression knockdown using siRNA, which has previously been demonstrated [6], was achieved using Stealth siRNA technology (Invitrogen, USA). RNAi conditions were optimized using Invitrogen's Green BLOCK-iT Fluorescent Oligo. THP-1 cells (5×10^5^ cells) were transfected with 200 pmole of each of 3 HDAC1 Stealth Select siRNA (HSS104725, HSS104726, HSS104727; Invitrogen, USA) or HDAC2 Stealth Select siRNA (HSS104728, HSS104729, HSS104730; Invitrogen, USA) using Lipofectamine RNAiMAX (Invitrogen, USA) in Opti-MEM. Negative Control siRNA #1 (Ambion, USA) was used as control. Twenty four hours after transfection, cells were infected with cell-free GFP-expressing *A. phagocytophilum* (MOI 10∶1) and samples were taken at 24 hours post-infection for determination of HDAC silencing effect on *A. phagocytophilum* infection by qPCR or by flow-cytometry as described above.

### Statistical methods

Statistical analysis was carried out using 2-sided Student's t-test for comparison means, where a p value <0.05 was considered significant. Error bars used throughout indicate standard error of the mean.

## Supporting Information

Figure S1 HDAC expression and activity is increased during infection with different isolates of *A. phagocytophilum*. HL-60 cells were infected with Webster, Slovenia and HZ strains of *A. phagocytophilum* for 48 hours. (A) Changes in the amount of HDAC1 with infection were determined by immunoblotting using an HDAC1-specific antibody. β-actin was used as a control. HDAC1 band intensity was determined by densitometric analysis and HDAC1 protein expression level changes with respect to uninfected cells were calculated. Samples were normalized for β-actin content. (B) HL-60 cells (HL) uninfected or infected with Webster (W), Slovenia (S) and HZ strains (HZ) of *A. phagocytophilum* were incubated for 24 h with and without 400 nM TSA. Histones were acid-extracted and acetylated and methylated H3 were detected by immunoblotting. Changes in the H3 acetylation and methylation patterns were determined by densitometric analysis of the immunoblot bands, and expressed as fold-change after TSA treatment with respect to the corresponding untreated control.
(0.44 MB TIF)

Table S1 Genes and primers used in this study.
(0.03 MB DOC)
