# Supplementary figures and images for: Epigenetic Silencing of Host Cell Defense Genes Enhances Intracellular Survival of the Rickettsial Pathogen Anaplasma phagocytophilum
Source: PLoS Pathog. 2009 Jun 19;5(6):e1000488. doi: 10.1371/journal.ppat.1000488 (PMC2694362; doi:10.1371/journal.ppat.1000488)

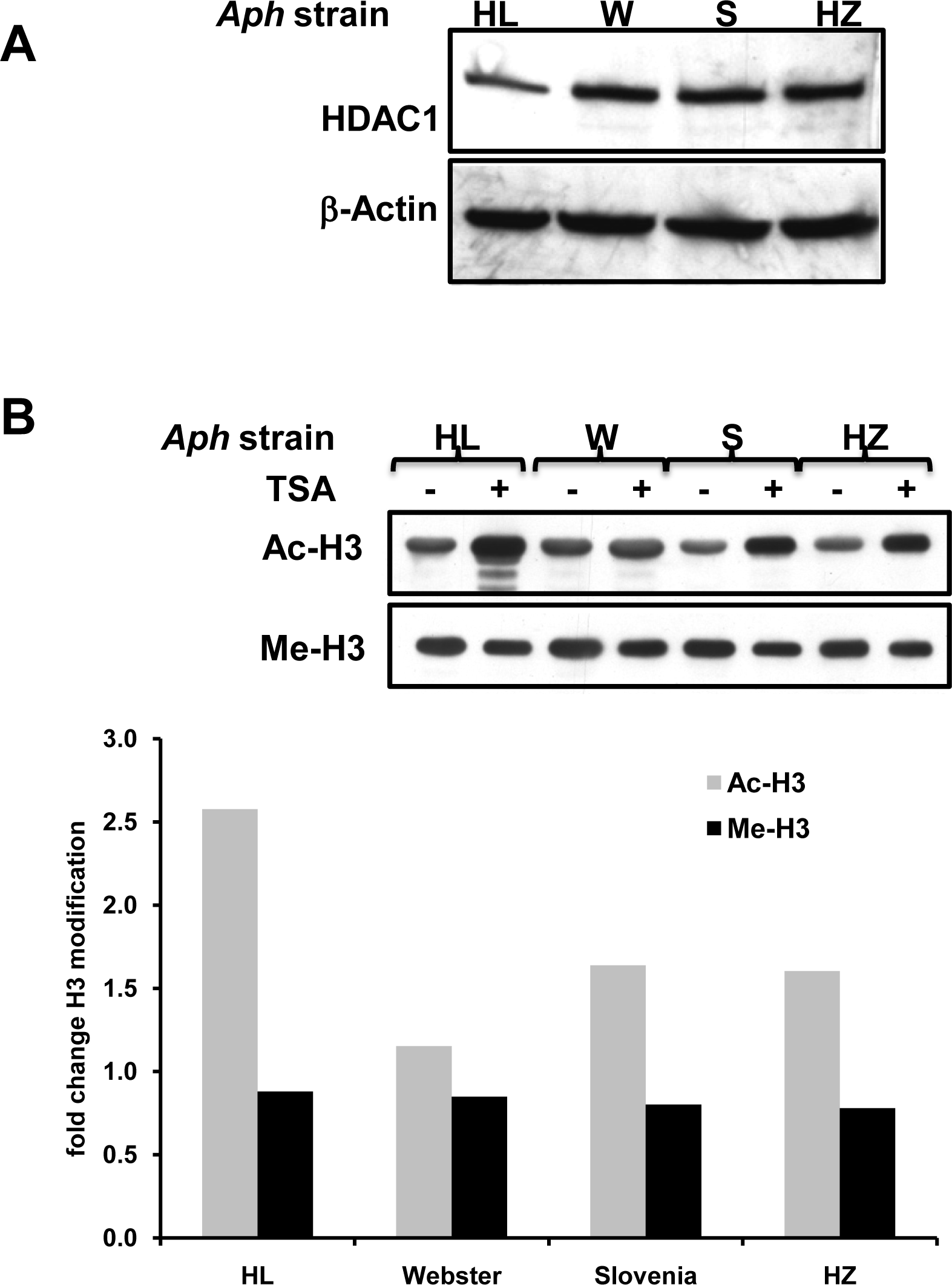

Supplement: Figure S1 — HDAC expression and activity is increased during infection with different isolates of A. phagocytophilum. HL-60 cells were infected with Webster, Slovenia and HZ strains of A. phagocytophilum for 48 hours. (A) Changes in the amount of HDAC1 with infection were determined by immunoblotting using an HDAC1-specific antibody. β-actin was used as a control. HDAC1 band intensity was determined by densitometric analysis and HDAC1 protein expression level changes with respect to uninfected cells were calculated. Samples were normalized for β-actin content. (B) HL-60 cells (HL) uninfected or infected with Webster (W), Slovenia (S) and HZ strains (HZ) of A. phagocytophilum were incubated for 24 h with and without 400 nM TSA. Histones were acid-extracted and acetylated and methylated H3 were detected by immunoblotting. Changes in the H3 acetylation and methylation patterns were determined by densitometric analysis of the immunoblot bands, and expressed as fold-change after TSA treatment with respect to the corresponding untreated control. (0.44 MB TIF) [file ppat.1000488.s001.tif]
