# Supplementary material for: Epigenetic Silencing of Host Cell Defense Genes Enhances Intracellular Survival of the Rickettsial Pathogen Anaplasma phagocytophilum
Source: PLoS Pathog. 2009 Jun 19;5(6):e1000488. doi: 10.1371/journal.ppat.1000488 (PMC2694362; doi:10.1371/journal.ppat.1000488)
Supplement: Table S1 — Genes and primers used in this study. (0.03 MB DOC) [file ppat.1000488.s002.doc]

Table S1. Genes and primers used in this study.

|  | **Symbol** | **Name** | **Primers** |
| --- | --- | --- | --- |
| Defense genes | *ACP2* | acid phosphatase 2 | ACP2-eF ATACAGCCAAGGCCACAATC  ACP2-eR GACTTCCTTCGCCTCACAGA |
|  | *ACP6* | acid phosphatase 6 | ACP6-eF ATAGCTGGGGGTTCCACTCT  ACP6-eR AAAATGGTGCAGGTCGTGTT |
|  | *AZU1* | azurocidin 1 | AZU1-eF CTGCTGATGGAAAACGTCTG  AZU1-eR CAGCTGCTTCCAAAGCCA |
|  | *CAMP* | cathelicidin antimicrobial peptide | CAMP-eF GGGCACACTGTCTCCTTCAC  CAMP-eR TCGGATGCTAACCTCTACCG |
|  | *CAT* | catalase | CAT-eF ACGGGGCCCTACTGTAATAA  CAT-eR AGATGCAGCACTGGAAGGAG |
|  | *CTSA* | cathepsin A | CTSA-eF GAGACGGCGGTACTGTAAGTTT  CTSA-eR TCCACCTACCTCAACAACCC |
|  | *CTSC* | cathepsin C | CTSC-eF CCTTGCTGCCCTCTTCTTTA  CTSC-eR ATTCTGGCCATTTCACCATC |
|  | *CTSG* | cathepsin G | CTSG-eF AAGATACGCCATGTAGGGGC  CTSG-eR TTCTGCTGGCCTTTCTCCTA |
|  | *DCD* | dermcidin | DCD-eF TAACCCTGGGTCTTCACCTG  DCD-eR CCCTGGTCTGTGCCTATGAT |
|  | *DEFA1* | defensin, alpha 1 | DEFA1-eF GGAGAATGGCAGCAAGGAT  DEFA1-eR AGACCTGGGACAGAGGACTG |
|  | *DEFA4* | defensin, alpha 4 | DEFA4-eF TGGAGGGCTACCAAGAGAAT  DEFA4-eR GTCTGCCCTCTCTGCTCG |
|  | *DEFA5* | defensin, alpha 5 | DEFA5-eF GGACTCACGGGTAGCACAAC  DEFA5-eR CCTTTGCAGGAAATGGACTC |
|  | *DEFA6* | defensin, alpha 6 | DEFA6-eF GACCTTCTGCAATGGCAAGT  DEFA6-eR AGGACTTTGCCGTCTCCTTT |
|  | *DEFB1* | defensin, beta 1 | DEFB1-eF GGGCAGGCAGAATAGAGACA  DEFB1-eR TTTTGTCTGAGATGGCCTCA |
|  | *DEFB4* | defensin, beta 4 | DEFB4-eF GCAGGTAACAGGATCGCCTA  DEFB4-eR ATCAGCCATGAGGGTCTTGT |
|  | *ELA2* | elastase 2 | ELA2-eF CACGTTGGCGTTGATGGT  ELA2-eR GCATCTTCGAAAACGGCTAC |
|  | *EPX* | eosinophil peroxidase | EPX-eF GCTAAGCTGTGCCAAATTCC  EPX-eR CTCTCAACATGCAACGAAGC |
|  | *GNLY* | granulysin | GNLY-eF CGCAGCATTGGAAACACTT  GNLY-eR GACCAAAACACAGGAGCTGG |
|  | *HAMP* | hepcidin antimicrobial peptide | HAMP-eF CTCCTTCGCCTCTGGAACAT  HAMP-eR AGTGGCTCTGTTTTCCCACA |
|  | *LYZ* | lysozyme | LYZ-eF ACAAGCTACAGCATCAGCGA  LYZ-eR GTAATGATGGCAAAACCCCA |
|  | *MPO* | myeloperoxidase | MPO-eF TCCCGAAGTAAGAGGGTGTG  MPO-eR CCCTGTCTCCTCACCAACC |
|  | *NOS2A* | nitric oxide synthase 2A | NOS2A-eF TCATTCTGCTGCTTGCTGAG  NOS2A-eR CAACAATGTGGAGAAAGCCC |
|  | *PRTN3* | proteinase 3 | PRTN3-eF GTGGATCAAGGTGCCTCC  PRTN3-eR GTGCTGCTGGCCTTGCT |
| Up-regulated controls | *IL8* | Interleukin 8 | IL8-eF ATGACTTCCAAGCTGGCCG  IL8-eR CTCCACAACCCTCTGCACC |
|  | *FTH* | ferritin heavy chain | FTH-eF CGACCGCGTCCACCTCG  FTH-eR CTTTCATTATCACTGTCTCCC |
| Housekeeping genes | *B2M* | Beta-2-microglobulin | B2M-eF TCTCTGCTGGATGACGTGAG  B2M-eR TAGCTGTGCTCGCGCTACT |
|  | *HPRT1* | Hypoxanthine phosphoribosyltransferase 1 | HPRT1-eF ACCCTTTCCAAATCCTCAGC  HPRT1-eR GTTATGGCGACCCGCAG |
|  | *RPL13A* | Ribosomal protein L13a | RPL13A-eF GGCCCAGCAGTACCTGTTTA  RPL13A-eR AGATGGCGGAGGTGCAG |
|  | *GAPDH* | Glyceraldehyde-3-phosphate dehydrogenase | GAPDH-eF AATGAAGGGGTCATTGATGG  GAPDH-eR AAGGTGAAGGTCGGAGTCAA |
|  | *ACTB* | Actin, beta | ACTB-eF GTTGTCGACGACGAGCG  ACTB-eR GCACAGAGCCTCGCCTT |
| HDACs | *HDAC1* | histone deacetylase 1 | HDAC1-eF CATCTCCTCAGCATTGGCTT  HDAC1-eR CGAATCCGCATGACTCATAA |
|  | *HDAC2* | histone deacetylase 1 | HDAC2-eF ATGAGGCTTCATGGGATGAC  HDAC2-eR ATGGCGTACAGTCAAGGAGG |
|  |  |  |  |
